# Endoscopic ultrasonography-guided gastroenterostomy versus surgical gastrojejunostomy for palliation of malignant gastric outlet obstruction (ENDURO): study protocol for a randomized controlled trial

**DOI:** 10.1186/s13063-023-07522-7

**Published:** 2023-09-25

**Authors:** Janine B. Kastelijn, Yorick L. van de Pavert, Marc G. Besselink, Paul Fockens, Rogier P. Voermans, Roy L. J. van Wanrooij, Thomas R. de Wijkerslooth, Wouter L. Curvers, Ignace H. J. T. de Hingh, Marco J. Bruno, Bas Groot Koerkamp, Gijs A. Patijn, Alexander C. Poen, Jeanin E. van Hooft, Akin Inderson, J. Sven D. Mieog, Jan-Werner Poley, Alderina Bijlsma, Daan J. Lips, Niels G. Venneman, Robert C. Verdonk, Hendrik M. van Dullemen, Frederik J. H. Hoogwater, Geert W. J. Frederix, I. Quintus Molenaar, Paco M. J. Welsing, Leon M. G. Moons, Hjalmar C. van Santvoort, Frank P. Vleggaar, for the Dutch Pancreatic Cancer Group

**Affiliations:** 1 Department of Gastroenterology and Hepatology, University Medical Center Utrecht, Utrecht, the Netherlands; 2 Department of Surgery, Amsterdam UMC, Location University of Amsterdam, Amsterdam, the Netherlands; 3 Cancer Center Amsterdam, Amsterdam, the Netherlands; 4 Department of Gastroenterology and Hepatology, Amsterdam UMC, Location University of Amsterdam, Amsterdam, the Netherlands; 5 Department of Gastroenterology and Hepatology, Amsterdam UMC, Location Vrije Universiteit, Amsterdam, the Netherlands; 6 Department of Gastrointestinal Oncology, Netherlands Cancer Institute, Amsterdam, the Netherlands; 7 Department of Gastroenterology and Hepatology, Catharina Hospital, Eindhoven, the Netherlands; 8 Department of Surgery, Catharina Hospital, Eindhoven, the Netherlands; 9 Department of Gastroenterology and Hepatology, Erasmus University Medical Center, Rotterdam, the Netherlands; 10 Department of Surgery, Erasmus University Medical Center Cancer Institute, Rotterdam, the Netherlands; 11 Department of Surgery, Isala Clinics, Zwolle, the Netherlands; 12 Department of Gastroenterology and Hepatology, Isala Clinics, Zwolle, the Netherlands; 13 Department of Gastroenterology and Hepatology, Leiden University Medical Center, Leiden, the Netherlands; 14 Department of Surgery, Leiden University Medical Center, Leiden, the Netherlands; 15 Department of Gastroenterology and Hepatology, Maastricht University Medical Center+, Maastricht, the Netherlands; 16 Department of Gastroenterology and Hepatology, Martini Hospital, Groningen, the Netherlands; 17 Department of Surgery, Medisch Spectrum Twente, Enschede, the Netherlands; 18 Department of Gastroenterology and Hepatology, Medisch Spectrum Twente, Enschede, the Netherlands; 19 Department of Gastroenterology and Hepatology, St. Antonius Hospital, Nieuwegein, the Netherlands; 20 Department of Gastroenterology and Hepatology, University Medical Center Groningen, University of Groningen, Groningen, the Netherlands; 21 Department of Surgery, University Medical Center Groningen, University of Groningen, Groningen, the Netherlands; 22 Julius Center for Health Sciences and Primary Care, University Medical Center Utrecht, Utrecht, the Netherlands; 23 Department of Surgery, St. Antonius Hospital, Nieuwegein, the Netherlands; 24 Department of Surgery, University Medical Center Utrecht, Utrecht, the Netherlands; 25 Division of Internal Medicine and Dermatology, University Medical Center Utrecht, Utrecht, the Netherlands

**Keywords:** Gastric outlet obstruction, Malignancy, Gastroenterostomy, Gastrojejunostomy, Endoscopic ultrasonography, Surgery, Clinical trial

## Abstract

**Background:**

Malignant gastric outlet obstruction (GOO) is a debilitating condition that frequently occurs in patients with malignancies of the distal stomach and (peri)ampullary region. The standard palliative treatment for patients with a reasonable life expectancy and adequate performance status is a laparoscopic surgical gastrojejunostomy (SGJ). Recently, endoscopic ultrasound-guided gastroenterostomy (EUS-GE) emerged as a promising alternative to the surgical approach. The present study aims to compare these treatment modalities in terms of efficacy, safety, and costs.

**Methods:**

The ENDURO-study is a multicentre, open-label, parallel-group randomized controlled trial. In total, ninety-six patients with gastric outlet obstruction caused by an irresectable or metastasized malignancy will be 1:1 randomized to either SGJ or EUS-GE. The primary endpoint is time to tolerate at least soft solids. The co-primary endpoint is the proportion of patients with persisting or recurring symptoms of gastric outlet obstruction for which a reintervention is required. Secondary endpoints are technical and clinical success, quality of life, gastroenterostomy dysfunction, reinterventions, time to reintervention, adverse events, quality of life, time to start chemotherapy, length of hospital stay, readmissions, weight, survival, and costs.

**Discussion:**

The ENDURO-study assesses whether EUS-GE, as compared to SGJ, results in a faster resumption of solid oral intake and is non-inferior regarding reinterventions for persistent or recurrent obstructive symptoms in patients with malignant GOO. This trial aims to guide future treatment strategies and to improve quality of life in a palliative setting.

**Trial registration:**

International Clinical Trials Registry Platform (ICTRP): NL9592. Registered on 07 July 2021.

**Supplementary Information:**

The online version contains supplementary material available at 10.1186/s13063-023-07522-7.

## Administrative information

The numbers in curly brackets in this protocol refer to SPIRIT checklist item numbers. The order of the items has been modified to group similar items (see http://www.equator-network.org/reporting-guidelines/spirit-2013-statement-defining-standard-protocol-items-for-clinical-trials/).

**Table:** 

Title {1}	Endoscopic ultrasonography-guided gastroenterostomy versus surgical gastrojejunostomy for palliation of malignant gastric outlet obstruction (ENDURO): study protocol for a randomized controlled trial
Trial registration {2a and 2b}.	International Clinical Trials Registry Platform (ICTRP): NL9592, registration date: 2021–07-07
Protocol version {3}	Version 4; date: 2022–01-10
Funding {4}	This study is funded by KWF Dutch Cancer Society
Author details {5a}	See page 13
Name and contact information for the trial sponsor {5b}	Frank P. Vleggaar, MD, PhD UMC Utrecht Department of Gastroenterology & Hepatology Heidelberglaan 100 3584CX, Utrecht The Netherlands + 31 88 75 593 38 f.vleggaar@umcutrecht.nl
Role of sponsor {5c}	The study sponsor is responsible for the study design, collection, management, analysis and interpretation of the data, writing of the report, and the decision to submit the report for publication. The study funder approved the study design.

## Introduction

### Background and rationale {6a}

Malignant gastric outlet obstruction (GOO) frequently occurs in patients with advanced malignancies of the stomach or (peri)ampullary region, due to mechanical obstruction of the distal stomach or duodenum [1, 2]. In patients diagnosed with pancreatic adenocarcinoma, 15–20% will develop GOO [3]. Depending on the severity of obstruction, symptoms may vary from early satiety, nausea, vomiting, to the inability to tolerate oral intake [2, 4]. These symptoms may have a significant impact on a patient’s quality of life [5].

Traditionally, two treatment modalities exist for malignant GOO. Enteral stenting (ES) is characterized by a fast relieve of symptoms. However, this comes at the cost of high rates of reinterventions due to stent obstruction in up to 30% of cases [6]. ES is therefore recommended in patients with an estimated survival of less than two months [7]. Laparoscopic surgical gastrojejunostomy (SGJ), on the other hand, is distinguished by its high success rate and low rate of reinterventions, but post-operative course may be complicated by significant morbidity, such as gastroparesis [7]. Hence, this procedure is recommended in patients with an estimated survival of more than 2 months and an adequate performance status [7].

A third treatment option was recently introduced: endoscopic ultrasonography-guided gastroenterostomy (EUS-GE), in which a lumen-apposing metal stent (LAMS) is positioned endoscopically between the stomach and a jejunal loop distal to the obstruction. The LAMS creates an alternative route for food to bypass the obstruction [8, 9]. EUS-GE seems to combine the advantages of both SGJ and ES [10]. Previous studies have shown a similar short time to resumption of oral intake after EUS-GE compared with ES [11–13]. Moreover, EUS-GE also seems to be associated with similar low reintervention rates as seen after SGJ [14–18]. However, EUS-GE is technically demanding and, when performed by inexperienced hands, might lead to adverse events (AEs) such as misdeployment of the LAMS, resulting in jejunal perforation and subsequent peritonitis [19–21]. Before EUS-GE can be adopted as standard treatment for malignant GOO, efficacy and safety should be assessed in a randomized trial. The aim of the ENDURO-study is to compare EUS-GE with SGJ in terms of effectiveness, safety, and costs.

### Objectives {7}

The primary objective of the study is to compare the effect of EUS-GE and SGJ on patients’ short- and long-term ability to eat. This is specified as two co-primary endpoints: time to resumption of oral intake and reinterventions for persistent or recurrent symptoms of GOO within 6 months of follow-up.

### Trial design {8}

This study is a multicentre, open-label, parallel-group randomized controlled trial. The trial assesses two co-primary outcomes: (1) the superiority endpoint time to oral intake of soft solids and (2) the non-inferiority endpoint persistent or recurrent GOO symptoms requiring reintervention. Participants will be 1:1 randomized to either SGJ (standard arm) or EUS-GE (experimental arm).

## Methods: participants, interventions, and outcomes

### Study setting {9}

The ENDURO-study is a nationwide multicentre trial in which seventeen Dutch hospitals will participate. Of these, six are academic and eleven are community teaching hospitals. The study is initiated by the University Medical Centre Utrecht and developed in collaboration with the Dutch Pancreatic Cancer Group (DPCG).

### Eligibility criteria {10}

#### Inclusion criteria

- Adult patients with symptomatic malignant gastric outlet obstruction, presenting with nausea, vomiting and/or inability to eat
- Gastric Outlet Obstruction Scoring System (GOOSS) Score of 0 (no oral intake) or 1 (liquids only) [22]
- Obstruction due to irresectable or metastatic malignancy without curative treatment options
- Radiologically or endoscopically confirmed gastric outlet obstruction
- Location of obstruction extending from the pyloric region to the distal duodenum (third part)
- Both treatments (SGJ and EUS-GE) are technically and clinically feasible
- Ability to provide written informed consent.

#### Exclusion criteria

- Radiological or clinical suspicion of other strictures or obstructions along the gastrointestinal tract (distal of the ligament of Treitz), with small intestinal dilation/ileus
- Cancer extending into the distal region or corpus of the stomach or around the ligament of Treitz
- Duodenal tube feeding is not tolerated, despite adequate position of the tube
- Altered anatomy after previous gastric, periampullary, or duodenal surgery
- Previous SGJ as palliative treatment for the same condition
- Inability to undergo surgery or upper endoscopy due to severe comorbidities (including large-volume ascites)
- World Health Organization (WHO) performance status of 4 (in bed 100% of time) [23]
- Uncorrectable coagulopathy, defined by INR > 1.5 or platelets < 50 × 10^9^/L

EUS-GE is technically demanding. To secure patient safety and avoid inclusion of a learning curve in this study, EUS-GE will only be performed by trained advanced endoscopists, with sufficient EUS-GE experience. Since SGJ is regarded as a standard procedure, no learning curve is expected. Enrolment criteria for participating centres are described in Additional file 1.

### Who will take informed consent? {26a}

If patients are eligible, the treating physician, research nurse, or study coordinator will explain the study and provide the patient information folder (PIF). After the patient has had the opportunity to ask additional questions and has had sufficient time to decide about participation, written informed consent for the ENDURO-study will be obtained by one of the above-mentioned persons.

### Additional consent provisions for collection and use of participant data and biological specimens {26b}

This trial does not involve collecting biological specimens.

## Interventions

### Explanation for the choice of comparators {6b}

EUS-GE will be compared with SGJ, because there is substantial uncertainty about the preferred treatment strategy in patients with malignant GOO who would qualify for SGJ in current practice, based on expected survival of more than 2 months and adequate performance status. Patients in whom both SGJ and EUS-GE are considered technically and clinically feasible can be included in the ENDURO-study.

### Intervention description {11a}

#### Investigational treatment: EUS-GE

The EUS-GE procedure has been standardized and protocolized in a Standard Operating Procedure (SOP) during several EUS-GE collaborators meetings, to assure uniformity. In Additional file 2, a detailed description is provided of pre- and postprocedural care. Preferentially, EUS-GE should be performed by two advanced endoscopists. A 20-mm LAMS will be used. The procedure is performed under deep sedation with propofol. Roughly, the EUS-GE procedure will be performed as follows:

A gastroscope is introduced proximally to the stenosis. Residual gastric contents will be removed. If not yet placed, a feeding tube or nasobiliary drain is placed distal to the stenosis through the working channel. The endoscope is then removed, followed by the introduction of a linear array echoendoscope into the stomach. With the echoendoscope, the jejunal or duodenal loop is identified by flushing saline mixed with a small amount of dye (indigo carmine) into the post-stenotic duodenal-jejunal loop. The endoscopist may choose to use additional fluoroscopy to evaluate the correct position of the potential place of puncture before creating the fistula tract.

The direct puncture technique will be used. The loop is punctured directly using an electrocautery-enhanced delivery system. The distal flange of the 20 × 10 mm LAMS (Hot AXIOS™ Stent, Boston Scientific Corporation) is deployed, followed by traction of the distal flange against the wall of the small intestine. The proximal flange is deployed and pushed out of the working channel during gentle retraction of the echoendoscope. Position and passage of the stent is confirmed by backflow of the indigo-coloured saline from the small intestine back into the stomach. The stent will be left to expand naturally, without immediate balloon dilation of the stent. All tubes will be removed immediately after EUS-GE is completed.

#### Comparator: SGJ (standard treatment)

The centre where SGJ is performed will be determined in multidisciplinary team meetings. To ensure uniformity, SGJ is standardized as well. A description of pre- and postprocedural care is provided in Additional file 2. The procedure will be performed as follows:

SGJ is performed laparoscopically. The anastomosis is positioned on the anterior or posterior side of the stomach. The exact position depends on tumour localization (antrum or antrum/corpus). An antecolic side-to-side gastrojejunostomy is created with or without separation of the omentum (without a Roux-Y reconstruction). The anastomosis is created with a 60-mm stapler (e.g. Endo GIA™ (Medtronic, Inc.)) and closed with a V-loc™ (Medtronic, Inc.) 3–0 suture or similar wound closure device. The length of the biliary limb (distance between bile duct and gastrojejunostomy) must be at least 50 cm. A surgical jejunostomy to allow enteral tube feeding is not routinely constructed. A tattoo may be placed in the efferent limb of the gastrojejunostomy to facilitate correct endoscopic placement of a nasojejunal feeding tube. If placed preoperatively, the nasojejunal tube will be removed. According to medical protocol, all patients will receive a nasogastric tube after SGJ.

Since SGJ is regarded a routine procedure, no learning curve is expected. SGJ will be performed in centers with experience in upper gastrointestinal or hepatopancreatobiliary surgery, according to usual healthcare pathways.

### Criteria for discontinuing or modifying allocated interventions {11b}

#### Excessive gastric residual volume before EUS-GE/SGJ

Occasionally, an excessive volume of gastric secretions and food remnants remain prior to EUS-GE or SGJ, preventing the safe administration of sedatives. When this situation arises, EUS-GE or SGJ will be rescheduled after additional extensive gastric decompression. This situation will not be considered technical failure.

#### EUS-GE failure

Patients are allowed to cross-over to the SGJ arm when EUS-GE attempt results in technical failure or the development of complications. Explicit informed consent to convert to SGJ in this case will be obtained beforehand.

### Strategies to improve adherence to interventions {11c}

Not applicable: it concerns a one-step procedure.

### Relevant concomitant care permitted or prohibited during the trial {11d}

In case of concomitant biliary obstruction, biliary intervention will be performed according to standard of care. Biliary interventions will not be performed in the same session as EUS-GE or SGJ.

### Provisions for post-trial care {30}

#### Persistent or recurrent symptoms after EUS-GE/SGJ

In case of persistent symptoms of gastric outlet obstruction (nausea, vomiting > 2 times during 24 h, inability to tolerate at least soft solid oral intake) after EUS-GE or SGJ, patients will be treated with a nothing per mouth regimen, nasogastric tube, and prokinetics (e.g. metoclopramide, domperidone, or erythromycin). If limited or no oral intake at postprocedural day five persists (GOOSS 0–1), a jejunal feeding tube will be placed under endoscopic guidance, after an intestinal ileus is excluded (distended abdomen, absent or high-pitched peristalsis, no passage of flatus or faeces). Endoscopic tube placement enables simultaneous assessment of anastomotic patency, and, in case of stent dysfunction or obstruction of the anastomosis, additional treatment. When feeding over a jejunal tube is not tolerated, parenteral nutrition will be initiated. Additional placement of a percutaneous endoscopic or radiologic gastrostomy will be left at the discretion of the treating physician.

In case of recurrent symptoms of gastric outlet obstruction—i.e. recurrence of nausea, vomiting, and inability to tolerate at least soft solid oral intake—upper endoscopy is advised.

Upper endoscopy, in addition to above-mentioned situations, or radiologic imaging will be performed at indication, e.g. in case of suspected gastrointestinal bleeding or perforation.

#### Escape intervention

Re-endoscopy through the LAMS is not allowed within the first 6 weeks after placement, since the fistula tract might not be fully matured yet.

If a patient had additional tube feeding prior to EUS-GE or SGJ, this is discontinued after the procedure in order to allow a fair chance of accepting oral intake. If necessary, tube feeding may be restarted, only if the patient has proven not to be able to maintain adequate oral intake, despite EUS-GE or SGJ.

#### Compensation for injury

The sponsor has a liability insurance which is in accordance with article 7 of the Dutch Medical Research Involving Human Subjects Act (WMO). This insurance provides cover for damage to research subjects through injury or death caused by the study. The insurance applies to the damage that becomes apparent during the study or within 4 years after the end of the study.

## Outcomes {12}

### Primary endpoints

Our main study parameter is the ability to eat. This will be measured with two co-primary endpoints, assessing the short and long-term effects:

- Time to oral intake of soft solids is defined as the number of days until a patient is able to tolerate soft solids (GOOSS ≥ 2**)** without vomiting;
- Persistent or recurrent GOO symptoms requiring reintervention is defined as any new intervention after EUS-GE or SGJ directed at improving or restoring nutritional intake, in case of persistent or recurrent obstructive symptoms of gastric outlet obstruction, such as nausea, vomiting and inability to tolerate oral intake (GOOSS 0–1).

In Additional file 3, a more detailed description is given of the two primary endpoints.

### Secondary endpoints

Secondary endpoints are described in detail in Additional file 3. We will assess the following secondary outcomes:

2. Technical success
3. Clinical success
4. Quality of life
5. Gastroenterostomy dysfunction
6. Reintervention
7. Time to reintervention
8. Adverse events
9. Time to start chemotherapy
10. Length of hospital stay
11. Readmission
12. Weight
13. Survival
14. Costs

## Participant timeline {13}

**Fig. 1:**
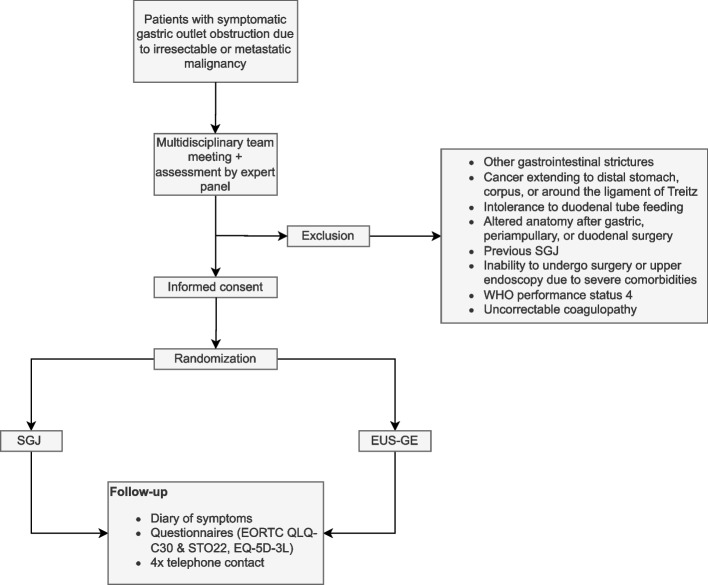
Study flowchart. *EUS-GE* endoscopic ultrasonography-guided gastroenterostomy, *SGJ* surgical gastrojejunostomy, *WHO* World Health Organization

See Fig. 1 and Table 1.

**Table 1:** SPIRIT diagram: schedule of enrolment, interventions, and assessments

	Study period											
	Eligibility assessment	Enrolment	Intervention	Post-allocation								
				Week				Month				
Timepoint	*-t*_*2*_	*-t*_*1*_	*t*_*0*_	*1*	*2*	*3*	*4*	*2*	*3*	*4*	*5*	*6*
**Enrolment:**												
** Eligibility screen**	X											
** Assessment by expert panel**	X											
** Discussion in clinical multidisciplinary meeting**	X											
** Informed consent **		X										
** Allocation**		X										
**Intervention:**												
***EUS-GE or SGJ***			X									
**Assessments:**												
***Baseline variables***		X										
***Procedural variables***			X									
***Intake and symptom diary***		X		Daily				Weekly				
***QoL surveys + diet & weight***		X			X		X		X			X
***Telephonic assessment of AEs and symptoms***					X		X		X			X

*AE* adverse event, *EUS-GE* endoscopic ultrasonography-guided gastroenterostomy, *SGJ* surgical gastrojejunostomy, *QoL* quality of life

## Sample size {14}

For the *primary endpoint* (i.e. time to ability to tolerate at least soft solids (GOOSS ≥ 2)), the median time to (re)gain the ability to eat soft solids was estimated at 3 days after laparoscopic SGJ and 1 day after EUS-GE, based on a retrospective study [14]. Assuming a reduction of time to oral intake from a median of 3 days to a median of 1 day for EUS-GE, a follow-up of 6 months, a two-sided alpha of 0.05 and an exponential survival curve, the estimated number of patients needed to obtain 90% power to detect a difference is 21 per arm as calculated using the POWER procedure in SAS (SAS Institute Inc., Cary, NC, USA).

The *co-primary endpoint* (i.e. persistent or recurrent symptoms of GOO, requiring a reintervention) can be assumed to occur in approximately 15% of patients for both treatments based on literature and experience [14, 15, 18, 24]. The reintervention risk for persistent or recurrent gastric outlet obstruction should not be significantly worse in the new intervention (EUS-GE) compared to the current standard treatment (SGJ). A non-inferiority margin of 20% (i.e. a maximum reintervention risk of 35%) is deemed acceptable, as limited access risk may be compensated with other benefits of EUS-GE (e.g. less invasive, less AEs, less costs). Also, the risk is comparable to the recurrence rate of duodenal stenting [25]. Using a reintervention risk of 15% for both groups, a non-inferiority margin of 20% and a one-sided alpha of 0.05, the estimated number of patients needed to show non-inferiority with 80% power is 44 participants per group, as calculated using the POWER procedure in SAS. As such a sample size of 44 per group appears to be sufficient to study both endpoints. To account for a 10% drop-out rate, we plan to include 96 patients in total (48 per group).

We will use a hierarchical testing procedure to avoid multiple testing: for EUS-GE to be the preferred treatment, we will first test the superiority of the primary endpoint (time to the ability to tolerate at least soft solids (GOOSS ≥ 2) and, if statistically significant, we will test the co-primary endpoint (persistent or recurrent symptoms of GOO requiring reintervention) for non-inferiority. When both tests are statistically significant EUS-GE is the preferred treatment and should be recommended to future patients with malignant GOO in a palliative setting.

## Recruitment {15}

Patients with symptomatic malignant GOO will present at the in- or outpatient department to discuss treatment options. Treating physicians (gastroenterologist, oncologist, or surgeon) will be aware of the ENDURO-study and screen these patients for eligibility. EUS-GE is a minimally invasive treatment compared to surgery, which is presumably preferred by patients for its less invasive nature. In the Netherlands, EUS-GE will only be performed in eligible patients within the ENDURO-study, since EUS-GE is an off-label, experimental treatment. This will potentially increase the enrolment rate. Eligible patients who refrain from participation will be offered the standard surgical treatment (SGJ).

An average recruitment rate of four inclusions per month is expected. We anticipate to complete patient enrolment within 2 years after trial initiation.

## Assignment of interventions: allocation

### Sequence generation {16a}

Patients will be randomly allocated with a 1:1 ratio to one of the study arms (EUS-GE or SGJ).

Randomization will be performed by block randomization, using random block size, stratified by WHO performance status (0–1 and 2–3) [23].

### Concealment mechanism {16b}

Not applicable: since the allocation sequence is computer-generated, no members of the study team have access to the sequence of randomization.

### Implementation {16c}

Generation of the randomization sequence and the process of randomization is performed electronically with Castor EDC (Ciwit B.V., Amsterdam, the Netherlands), a validated and General Data Protection Regulation (GDPR) compliant data management program. Randomization is performed by the study coordinator.

## Assignment of interventions: blinding

### Who will be blinded {17a}

Blinding of participants and physicians who perform the intervention is not feasible, as it concerns an endoscopic and a surgical treatment. However, after the completion of the trial, an outcome adjudication panel will be appointed that will be blinded for the intervention that has been performed. All panel members will evaluate the outcomes independently and consensus meetings will be held to discuss discrepancies.

### Procedure for unblinding if needed {17b}

Not applicable: blinding mechanisms will not be used in this trial.

## Data collection and management

### Plans for assessment and collection of outcomes {18a}

Data will be collected during a follow-up period of 6 months (24 weeks) after the intervention or until death.

#### Diary of symptoms

In order to measure the co-primary endpoints adequately, obstructive symptoms and intake will be registered on a daily basis during the first month after EUS-GE or SGJ as patient-reported outcomes. Four items will be scored as multiple-choice questions: diet tolerability, nausea, vomiting, and pain. The validated gastric outlet obstruction scoring system (GOOSS) will be used to score diet tolerability (Table 2) [22]. This score is designed to provide an objective grade to a patients’ ability to eat before and after gastric outlet obstruction procedures.

**Table 2:** Gastric Outlet Obstruction Scoring System (GOOSS) [22]

Score	Ability of oral intake
0	No oral intake possible
1	Only liquid intake possible
2	Ability to eat soft solids
3	Ability to eat a low-residue or full diet

An online or paper diary will be used to register these four questions daily, in order to capture the moment of improvement of food intake (primary outcome, time to oral intake of soft solids) and relief of symptoms, which is expected within the first month. After the first month, this will be done once a week. In addition, a patient will be asked to weigh him/herself and to specify oral intake at four prespecified time points during follow-up (at weeks 2, 4, 12, and 24).

#### Quality of life

Quality of life will be measured with two validated questionnaires: the European Organisation of Research and Treatment for Cancer (EORTC) QLQ-C30 core questionnaire supplemented with the disease-specific EORTC QLQ-STO22, to focus on quality of life related to obstructive symptoms [26, 27]. In addition, the short EQ-5D-3L questionnaire will be used to calculate quality-adjusted life years (QALYs) and use these outcomes for cost-effectiveness analyses [28]. Participants are asked to answer these questionnaires before treatment (baseline) and after 2, 4, 12, and 24 weeks.

#### Telephone contact

The coordinating investigator or research nurse will call the patient after 2, 4, 12, and 24 weeks, to verify oral intake, assess recurrence of obstructive symptoms, and evaluate whether any other adverse events have occurred.

### Plans to promote participant retention and complete follow-up {18b}

In order to adequately evaluate the number of days required to resume oral intake, participant diaries need to be filled out accurately and timely. Diary and survey progress can be viewed online through Castor EDC. If participants appear to be having difficulties in answering the diary or questionnaires, online reminders will be sent.

Questionnaires on quality of life, oral intake, and weight have to be answered on the same day as follow-up by telephone is scheduled. Participants will be reminded to fill out the questionnaires during the phone call.

### Data management {19}

Data will be extracted by authorized research group personnel, i.e. the local investigator, research nurse, or study coordinators, through electronic case report forms (eCRFs). These will be stored in Castor EDC. The eCRF will be checked for accuracy and completed where needed by the study coordinators. Castor EDC has an audit trail; changes and documentation within the eCRFs will be tracked. Questionnaires and diaries will also be distributed via Castor EDC, unless participants prefer to receive them on paper.

Data validation will be performed, with multiple checks on the completeness and consistency of the data. The audit trail will be saved.

### Confidentiality {27}

Collected data will be coded and pseudonymized. Every included patient will be given a unique study number. This study number will be linked to the included participant in a separate and secured file, to enable retrieval of the source data when needed.

Data management will be performed by the study coordinator, supported by a research nurse and the local internal data manager. Staff involved will be Good Clinical Practice (GCP) qualified. Only the research group personnel and monitor will have access to the source data and to the database.

### Plans for collection, laboratory evaluation, and storage of biological specimens for genetic or molecular analysis in this trial/future use {33}

Not applicable: no biological specimens will be used in this trial.

## Statistical methods

### Statistical methods for primary and secondary outcomes {20a}

Descriptive statistics will be used for patient characteristics. For continuous variables, means with standard deviations (SD) will be used for normally distributed variables, and medians with interquartile ranges (IQR) for variables with a skewed distribution. Categorical variables will be reported as frequencies and proportions (%).

A *p*-value below 0.05 will be considered statistically significant. No correction for multiple testing will be performed.

#### Primary endpoints

For the primary endpoint (time to the ability to tolerate at least soft solids (GOOSS ≥ 2)), Kaplan Meier survival curves will be constructed and differences between groups will be tested using the log-rank test. To control for the stratification factor (WHO performance status) used at randomization and other relevant prognostic factors, a Cox-proportional hazards model will be generated as primary analysis. The hazard ratio for treatment will be calculated with 95% confidence interval and tested for statistical significance at an alpha level of 0.05.

Patients will be censored at the end of follow-up (6 months) or death.

For the co-primary endpoint (persistent or recurrent GOO symptoms requiring reintervention), a logistic regression analysis will be performed corrected for the stratification factor used at randomization (WHO performance status) and other relevant prognostic factors. A risk difference with a 90% confidence interval will be constructed and the upper limit of this confidence interval (i.e. one-sided test with alpha of 0.05) will be compared against the non-inferiority limit. When this upper limit does not cross the non-inferiority limit, EUS-GE will be considered non-inferior regarding the co-primary endpoint.

#### Secondary endpoints

For binary endpoints (e.g. technical and clinical success, occurrence of (serious) adverse events ((S)AEs)), logistic regression analyses will be performed using the same covariates as for the analysis of the primary endpoints. For time-to-event endpoints (overall survival, time to recurrence, time to start of chemotherapy), a Cox proportional hazards model will be generated the same way as for the primary endpoint. Continuous secondary endpoints will be analysed using linear regression analysis, again using the same covariates. For longitudinally measured continuous outcomes, longitudinal analysis of covariance will be performed by estimating a linear mixed model with post-baseline QoL values as a dependent variable, a random intercept at subject level, and fixed effects for baseline QoL, treatment arm, and time.

Longitudinal analysis of covariance will be performed by estimating a linear mixed model with post-baseline QoL values as a dependent variable, a random intercept at the subject level, and fixed effects for baseline QoL, treatment arm, and time as well as the previously mentioned covariates. The interaction between treatment arm and time will also be added to this model. The effect of treatment will be tested by comparing the model with and without the treatment term(s). A similar analysis will be performed for other longitudinally measured continuous outcomes.

Economic consequences of EUS-GE and SGJ will be estimated from a societal perspective and related to patient outcome in terms of quality-adjusted life years (QALYs). Both costs and outcomes will be linked in a decision analytic model to extrapolate outcomes over longer time periods. Outcome measures—QALYs and incremental cost-effectiveness ratios (ICERs)—will be used for the cost-utility analysis. Probabilistic and deterministic analyses will be performed according to the Dutch guidelines for economic evaluations in healthcare.

### Interim analyses {21b}

No interim analysis is planned.

### Methods for additional analyses (e.g. subgroup analyses) {20b}

A competing risk analysis for the primary endpoint (time to the ability to tolerate at least soft solids (GOOSS ≥ 2)) will also be performed as a secondary analysis.

No subgroup analyses are planned.

### Methods in analysis to handle protocol non-adherence and any statistical methods to handle missing data {20c}

Both intention-to-treat and per-protocol analyses will be performed. Per protocol analyses will be performed in the groups that included patients who have received the allocated treatment (EUS-GE or SGJ).

In time-to-event analyses, missings/lost-to follow-up will be handled by censoring. As our repeated continuous outcomes are analysed using linear mixed models, which give unbiased results in the presence of missing data, we will not impute these outcomes. For our binary outcomes, we will use multiple imputation by chained equations when 10% of patients or more have missing outcome information.

### Plans to give access to the full protocol, participant-level data and statistical code {31c}

This study is registered in a publicly available prospective clinical trial registry before the first patient was included (International Clinical Trials Registry Platform (ICTRP) number NL9592). Access to participant-level data and the statistical code can be granted on request, since the data used in this trial is privacy-sensitive.

## Oversight and monitoring

### Composition of the coordinating centre and trial steering committee {5d}

The trial coordinator and principal investigator (PI) of the coordinating centre will run the trial day-to-day. They oversee the general progress and conduct of the study and are responsible for supervising the participating centres. They meet on a daily or weekly basis.

Every participating centre has a head investigator, who will present potential trial participants to the trial coordinator. The trial coordinator subsequently presents these potential trial participants to the expert panel. This panel consists of three EUS-GE-experienced endoscopists and three hepatopancreatobiliary surgeons. Based on several clinical and radiological characteristics, they determine whether potential participants meet the inclusion criteria. Only after approval of the expert panel, patients will be approached and asked for informed consent.

### Composition of the data monitoring committee, its role and reporting structure {21a}

No data monitoring committee (DMC) will be installed at the initiation of the study, because the (S)AE rates of EUS-GE are expected to be similar or lower compared to SGJ. The ENDURO expert panel will guard the safety of this study by reviewing the data on SAEs that have occurred. Any event which is considered Clavien-Dindo 3B or higher will be reviewed by the panel to evaluate its relation with the procedure [29]. After notifying the involved competent authority, the review panel will consider the establishment of a DMC in close cooperation with the institutional review board (IRB).

### Adverse event reporting and harms {22}

All AEs reported by the participant or observed by the investigator or the clinician will be recorded.

In the ENDURO-study, AEs are defined according to the ASGE lexicon for endoscopic adverse events: “an adverse event is one that prevents completion of the planned procedure and/or results in hospital admission, prolongation of existing hospital stay, another procedure, or subsequent medical consultation” [30]. AEs are graded according to the Clavien-Dindo classification of surgical complications (Table 3) [29].

**Table 3:** The Clavien-Dindo classification of surgical complications [29]

Grade	Definition
I	Any deviation from the normal postoperative course without the need for pharmacological treatment or surgical, endoscopic, and radiological interventions Allowed therapeutic regimens are: drugs as antiemetics, antipyretics, analgetics, diuretics, electrolytes, and physiotherapy. This grade also includes wound infections opened at the bedside
II	Requiring pharmacological treatment with drugs other than such allowed for grade I complications Blood transfusions and total parenteral nutrition are also included
III	Requiring surgical, endoscopic or radiological intervention
IIIa	Intervention not under general anaesthesia
IIIb	Intervention under general anaesthesia
IV	Life-threatening complication (including CNS complications) requiring IC/ICU management
IVa	Single organ dysfunction (including dialysis)
IVb	Multiorgan dysfunction
V	Death of a patient

*CNS* Central nervous system, *IC* Intermediate care, *ICU* Intensive care unit

SAEs are defined as AEs of Clavien-Dindo 3B (intervention under general anaesthesia) or higher. Because of the palliative setting of the study population, clinical deterioration and death are inevitable events. For that reason, reporting of SAEs to the IRB will be limited to the SAEs that are Clavien-Dindo 3B or higher *and* which are judged by the expert panel as probably or definitely related to EUS-GE or SGJ.

AEs will be extracted from the electronic health record, the symptom diary, or from one of the telephone follow-up calls.

### Frequency and plans for auditing trial conduct {23}

The investigational treatment (EUS-GE) carries little additional risks compared to the standard treatment (SGJ). Therefore, monitoring based on a low-risk classification will be applied. This will be performed by an independent body and is in agreement with national guidelines. A monitoring visitation consists of evaluation of the correct use of inclusion and exclusion criteria, source data review and verification, and appropriate documentation of SAEs.

Monitoring involves one initiation visit, one regular monitoring visit, and one close-out visit (remote or on-site) of each participating centre and of the coordinating centre.

### Plans for communicating important protocol amendments to relevant parties (e.g. trial participants, ethical committees) {25}

Substantial amendments that are likely to significantly affect the safety or physical or mental integrity of the participants of the trial, the scientific value of the trial, the conduct or management of the trial, or the quality or safety of any intervention used in the trial will be notified to the IRB. After approval of the IRB, these substantial amendments will be communicated to the involved investigators in the participating centres.

If the amendments could have any influence on the decision of participants to participate in the trial, these changes will be communicated to the participants as well.

## Dissemination plans {31a}

The results of this study will be published in a high-impact peer-reviewed scientific journal. Additionally, results will be submitted for presentation at national and international congresses,

Results will be presented at the website of the DPCG and disseminated via its social media channels. Results will also be presented to the patient organizations (Living with Hope Foundation), on their website, at educational events, or through other channels, as will be determined together with the patient associations.

## Discussion

The ENDURO-study is designed to answer the clinical question whether EUS-GE compared with laparoscopic SGJ results in faster resumption of solid oral intake with a non-inferior rate of reinterventions for persistent or recurrent obstructive symptoms in patients with malignant GOO within a palliative setting. This is the first published protocol describing an RCT with this comparison.

Currently, two clinical trials are recruiting patients who are randomly allocated to EUS-GE or ES. The primary endpoint of these trials is the rate of recurrence of GOO and reinterventions, respectively (NCT03259763 and NCT03823690). Our study includes patients who would qualify for SGJ, based on a more than 2 months expected survival and adequate performance status, in whom ES is considered a less suitable treatment option because of limited patency [31].

Four other randomized trials comparing EUS-GE with SGJ have started recently or will start recruiting patients (NCT05548114, NCT05564143, NCT05561907, and NCT05605327). Similar to the ENDURO-study, three trials assess time to oral intake or functional recovery as primary endpoint, but planned to recruit a smaller number of patients (NCT05548114, NCT05564143, and NCT05561907). One trial has procedure-related adverse events as its primary outcome (NCT05605327).

To date, seven cohort studies have been published that compared EUS-GE with SGJ [14–18, 32, 33]. In addition to the retrospective design, these studies are limited by small sample sizes and/or the use of different surgical and EUS-guided approaches. Especially two early studies may have been subject to inexperience with this new technique, resulting in a high rate of LAMS misdeployments (3 in 30 patients (10%) and 9 in 25 patients (36%)) [15, 17].

It is estimated that approximately 7 to 25 procedures are required to achieve proficiency in performing EUS-GE [19, 20]. To avoid the effect of a learning curve, EUS-GE will be performed by trained advanced endoscopists who demonstrated sufficient experience with the procedure and devices. In order to participate, the following criteria have to be met:

First, exclusively centres that have performed > 20 LAMS placements for other indications—such as drainage of pancreatic pseudocysts, walled-off necrosis, the biliary tract, or the gallbladder—are allowed to participate in this trial. Second, in case participating centres do not have sufficient experience in performing EUS-GE specifically (< 10 procedures), procedures will be performed with the direct proctoring of an expert endoscopist (> 20 EUS-GE performed). After the participating centre has performed at least 10 procedures *and* has received a positive evaluation of competency by the proctoring endoscopist, the centre is allowed to perform EUS-GE independently. Third, all participating centres are strongly advised to perform the procedure with two endoscopists, to guarantee maximum expertise and safety. See Additional file 1 for detailed enrolment criteria for participating centres.

The ENDURO-study is designed as a pragmatic trial to improve external validity. However, certain aspects of both procedures and postprocedural care were standardized for safety reasons and to avoid heterogeneity of the procedures and postprocedural care within and between the treatment arms (see paragraph on intervention description and Additional file 2).

An important remaining difference is that patients who will undergo SGJ will receive a nasogastric tube as this is routine practice in most hospitals in the Netherlands, whereas after EUS-GE patients will not receive a nasogastric tube, because it may result in dislocation of the LAMS. To prevent patients from not resuming oral intake due to the prolonged indwelling of a nasogastric tube, a protocol is adopted to ensure the tube will be removed when gastric residual volume is below a certain threshold (200–300 ml). In both arms, the enteric feeding tube will be removed during or directly after the procedure. These standardized steps allow for an accurate comparison of the time to resume oral intake in both treatment arms.

In the ENDURO-study, WHO performance status will be used as a stratification factor [23]. Several studies have shown that poor performance status is a prognostic factor for survival in patients with malignant GOO [34, 35]. We do not stratify by centre since this might negatively affect allocation concealment and balance between the two arms [36]. Furthermore, in some cases, patients allocated to EUS-GE will be transferred to other centres for logistical reasons or because some hospitals do not perform EUS-GE.

In short, the ENDURO-study compares the efficacy and safety of EUS-GE with SGJ in patients with malignant GOO, aiming to improve quality of life and guide future palliative treatment strategies.

## Trial status

The first patient was included and randomized on February 18, 2022. Currently (2023–04-12), 50 patients have been included and the accrual rate proceeds as scheduled. Protocol version 4 (2022–01-10) is being used. The last patient is expected to be included in the first half of 2024.

### Supplementary Information

**Additional file 1.** Criteria for participating centres. Eligibility criteria for hospitals to participate in the trial.

**Additional file 2.** Study procedures. A detailed description of pre and postprocedural care.

**Additional file 3.** Primary and secondary endpoints. A detailed explanation of primary and secondary endpoints.

## Data Availability

The coordinators of the ENDURO-study will have access to the final trial dataset. Any data required to support the protocol can be supplied on request.

## Abbreviations

AE: Adverse event
DMC: Data monitoring committee
DPCG: Dutch Pancreatic Cancer Group
eCRF: Electronic Case Report Form
EDC: Electronic Data Capture
ES: Enteral stent
EORTC: European Organisation of Research and Treatment for Cancer
EUS-GE: Endoscopic ultrasonography-guided gastroenterostomy
GDPR: General Data Protection Regulation
GCP: Good Clinical Practice
GOO: Gastric outlet obstruction
GOOSS: Gastric Outlet Obstruction Scoring System
ICER: Incremental cost-effectiveness ratio
ICTRP: International Clinical Trials Registry Platform
IRB: Institutional review board
IQR: Interquartile range
LAMS: Lumen-apposing metal stent
PI: Principal investigator
PIF: Patient information folder
SAE: Serious adverse event
SD: Standard deviation
SEMS: Self-expandable metallic stent
SGJ: Surgical gastrojejunostomy
SOP: Standard Operating Procedure
QALY: Quality-adjusted life-year
QoL: Quality of life
WHO: World Health Organization
WMO: Dutch Medical Research Involving Human Subjects Act
