# Supplementary material for: Endoscopic ultrasonography-guided gastroenterostomy versus surgical gastrojejunostomy for palliation of malignant gastric outlet obstruction (ENDURO): study protocol for a randomized controlled trial
Source: Trials. 2023 Sep 25;24:608. doi: 10.1186/s13063-023-07522-7 (PMC10518948; doi:10.1186/s13063-023-07522-7)
Supplement: Supplementary file 1 — Additional file 1. Criteria for participating centres. Eligibility criteria for hospitals to participate in the trial. [file 13063_2023_7522_MOESM1_ESM.pdf]

Additional file 1 – Enrolment criteria for participating centres

Participating centres will be enrolled as either 'experienced' or 'supervised' centres. A centre is considered an 'EUS-GE experienced centre' if it has performed at least 20 EUS-GE procedures. This number includes endoscopic ultrasound-guided transgastric ERCP (EDGE).
Centres not fulfilling the above-mentioned criterium, but with the ambition to perform EUS-GE and with experience in at least 20 LAMS placements for other indications (gallbladder, pancreatic collections, drainage of the biliary tract), may participate as 'supervised centre'.
A supervised centre that has performed less than ten EUS-GE placements will be directly proctored by an endoscopist of an experienced centre. In case a supervised centre has successfully completed 10 or more but less than 20 EUS-GE's, the centre is allowed to perform the procedure independently after approval of an EUS-GE experienced endoscopist. A 'supervised centre' will become an 'EUS-GE experienced centre' after having performed at least 20 EUS-GE's. Nevertheless, supervised centres are recommended to perform all of the EUS-GE procedures within the ENDURO-study under supervision, as this will guarantee sufficient training, optimal gain of experience and maximum safety.

Centres without ambition to perform EUS-GE will refer their patients to an 'EUS-GE experienced centre'. See table 1 for a schematic overview of the participating centre enrolment requirements.

Table 1 Participating centre enrolment requirements

	**Minimum enrolment prerequisite**	**Supervised centre**	**Unsupervised centre**	**Experienced centre**
**LAMS experience***	≥ 20	≥ 20	≥ 20	≥ 20
**EUS-GE experience***	-	0-10	11-20	> 20

* Number of procedures performed per participating centre. LAMS lumen-apposing metal stent; EUS-GE endoscopic ultrasonography-guided gastroenterostomy
