# Supplementary material for: Endoscopic ultrasonography-guided gastroenterostomy versus surgical gastrojejunostomy for palliation of malignant gastric outlet obstruction (ENDURO): study protocol for a randomized controlled trial
Source: Trials. 2023 Sep 25;24:608. doi: 10.1186/s13063-023-07522-7 (PMC10518948; doi:10.1186/s13063-023-07522-7)
Supplement: Supplementary file 2 — Additional file 2. Study procedures. A detailed description of pre and postprocedural care. [file 13063_2023_7522_MOESM2_ESM.pdf]

Additional file 2 – Preprocedural and postprocedural care

Investigational treatment: EUS-GE

Preprocedural measures

- Fasting: aim for an as empty as possible stomach, by 12 hours fasting for solid food, 6 hours fasting for liquids.
- Nasogastric tube: nasogastric tube placement to allow emptying of the stomach and limit gastric distension is obligatory prior to EUS-GE to reduce risk of aspiration of gastric contents. If indicated, plain abdominal radiography will be performed to verify an adequate position of the nasogastric tube.
- Jejunal feeding tube: if already placed for tube feeding, the tube will be left in situ to be used for flushing and filling the post-stenotic duodenal-jejunal loop during the EUS-GE placement.
- Antibiotics: One dose of prophylactic intravenous antibiotics, covering intra-abdominal infections will be administered 30 minutes prior to or during EUS-GE. For example, a single dose of ceftriaxone 2000 mg and metronidazole 500 mg, or an equivalent in accordance with local protocol based on local resistance patterns.
- Anticoagulants and antiplatelet drugs: In case vitamin K antagonists are used, INR needs to be < 1.5. Direct acting oral anticoagulants need to be discontinued 48 hours prior to the procedure. Antiplatelet monotherapy is allowed. In case of dual antiplatelet therapy, one of the two drugs needs to be discontinued five days prior to the procedure, in accordance with the intervention with high-bleeding risk guideline. Anticoagulants and/or antiplatelet agents are typically restarted 24 hours post-procedurally.

Postprocedural care

- Nasogastric tube or duodenal feeding tube: all tubes will be immediately removed after EUS-GE is completed. Tubes left in the gastrointestinal tract after EUS-GE pose a risk of stent dislodgement.
- Diet: the day of the intervention a clear liquid diet can be initiated. The next day this will be advanced as tolerated, to an easily digestible/low residue food diet, and if this is accepted well, to solid food (figure 1). Since patients and/or caregivers tend to feel anxious to restart oral intake, this should be clearly communicated and encouraged. A skilled dietician can be contacted upon

request to answer intake related questions.

- Antibiotics: No post-procedural antibiotics are administered, unless indicated.
- Discharge: when judged clinically possible.

Due to the limited prognosis of the research participants and the expected burden of recurrent endoscopic interventions, the LAMS will be left in place permanently.

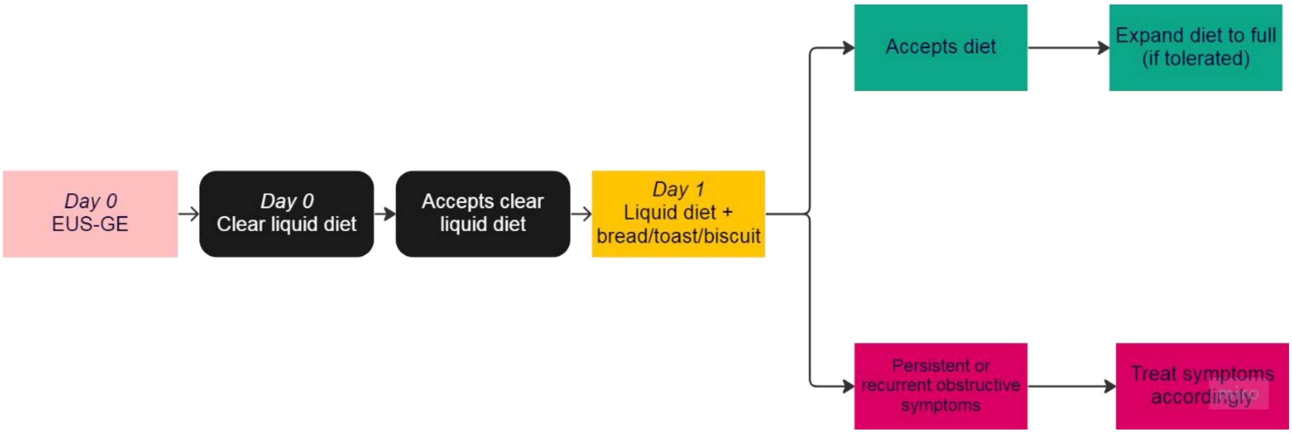

Fig. 1 Postprocedural care after EUS-GE

Comparator: SGJ (standard treatment)

Patients will be prepared for SGJ in a similar fashion to EUS-GE (see paragraph on EUS-GE preprocedural measures).

Postoperative care

- Nasogastric tube: According to medical protocol, all patients will receive a nasogastric tube after SGJ. The morning after the SGJ procedure, the nasogastric tube will be disconnected from the collection bag but remain in situ. After 4 hours gastric residual volume will be determined. When the residual volume is less than 200-300 ml, the tube will be removed. When the volume exceeds this limit, every 24 hours gastric residual volume will be determined. When the residue is less than 200-300 ml measured over four hours, the tube will be removed (figure 2).
- Nasojejunal feeding tube: A nasojejunal feeding tube will not routinely be placed in patients after SGJ. In case of apparent delayed gastric emptying, a nasojejunal feeding tube will be placed under endoscopic guidance.
- Diet: Patients will not commence oral intake unless the gastric residual volume is at an acceptable level (see nasogastric tube). However, a clear liquid diet is tolerated immediately after SGJ. When the gastric residual volume is acceptable, patients will start with a soft solid food diet. This will be advanced in a stepwise manner, eventually – if tolerated – to a full solid diet (figure 2).
- Antibiotics: No post-procedural antibiotics are administered, unless indicated.
- Discharge: when judged clinically possible.

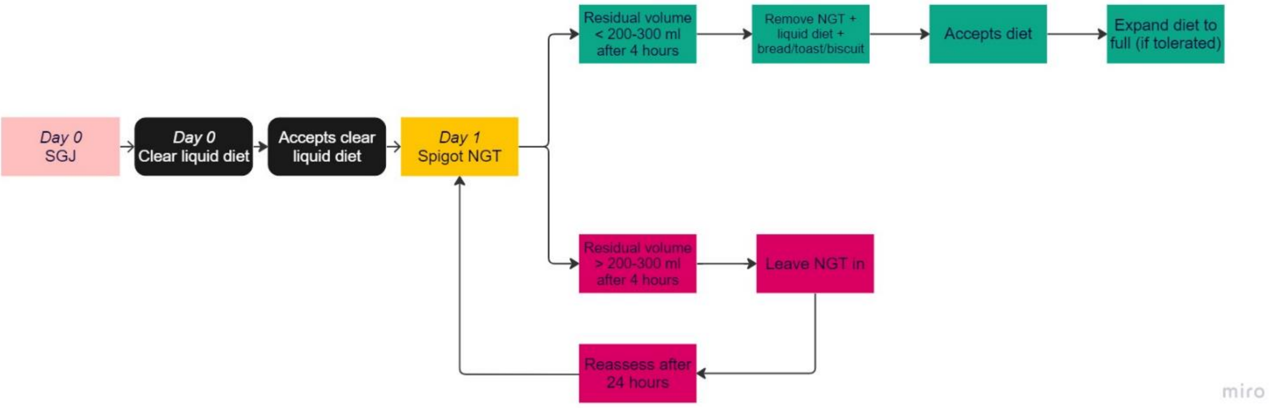

Fig. 2 Postoperative care after SGJ. NGT nasogastric tube
