# Supplementary material for: Endoscopic ultrasonography-guided gastroenterostomy versus surgical gastrojejunostomy for palliation of malignant gastric outlet obstruction (ENDURO): study protocol for a randomized controlled trial
Source: Trials. 2023 Sep 25;24:608. doi: 10.1186/s13063-023-07522-7 (PMC10518948; doi:10.1186/s13063-023-07522-7)
Supplement: Supplementary file 3 — Additional file 3. Primary and secondary endpoints. A detailed explanation of primary and secondary endpoints. [file 13063_2023_7522_MOESM3_ESM.pdf]

Additional file 3 – primary and secondary endpoints

Primary endpoints

1a. Time to oral intake of soft solids is defined as the number of days until a patient is able to tolerate soft solids without vomiting. Day of intervention is considered day 0. The ability to tolerate oral intake is assessed with the Gastric Outlet Obstruction Scoring System (GOOSS), which is depicted in table 1 [1].

Table 1 Gastric Outlet Obstruction Scoring System (GOOSS) [1]

Score	Ability of oral intake
**0**	No oral intake possible
**1**	Only liquid intake possible
**2**	Ability to eat soft solids
**3**	Ability to eat a low-residue or full diet

1b. Persistent or recurrent GOO symptoms requiring reintervention is defined as any new intervention after EUS-GE or SGJ directed at improving or restoring nutritional intake, in case of persistent or recurrent obstructive symptoms of gastric outlet obstruction, such as nausea, vomiting and inability to tolerate oral intake (GOOSS 0-1).

Reinterventions may be categorized as follows:

- Endoscopic procedures aimed at evaluating and, if applicable, treating anastomotic dysfunction, such as removing impacted food/tissue overgrowth, insertion of a second LAMS after migration of the first, or procedures aimed at creating an alternative route for food passage, such as placement of an enteral stent or creation of a percutaneous jejunostomy or venting gastrostomy, or placement of an oral feeding tube;
- Surgical procedures aimed at treating dysfunction of the gastroenteric anastomosis or creating alternative ways for food passage, such as creation of a percutaneous jejunostomy;
- Radiologic procedures to treat symptoms of gastric outlet obstruction, such as construction of a percutaneous gastrostomy or jejunostomy;

- Initiation of total parenteral nutrition.

Secondary endpoints

2. Technical success is defined as successful creation of a gastroenterostomy by means of the allocated technique (EUS-GE or SGJ). Successful (stent-in-stent) placement of a second Hot AXIOS™ stent during the same procedure after the initial attempt failed is also considered technically successful. If additional techniques, modalities or different type of stents (e.g. self-expandable metallic stents) were required, it will be regarded as technical failure.
3. Clinical success is defined as relief of symptoms and toleration of soft solids (GOOSS ≥ 2) without vomiting.
4. Gastroenterostomy dysfunction is defined as recurrence of obstructive symptoms (GOOSS 0-1) due to recurrence of GOO at the gastroenterostomy site after initial clinical success, confirmed endoscopically or radiographically.
5. Reintervention is defined as any radiologic, endoscopic or surgical intervention for an adverse event, persistent obstructive symptoms or recurrent obstructive symptoms, that is needed after EUS-GE or SGJ. This includes creating an alternative route to improve or restore adequate nutritional intake – either through placing a nasal feeding tube, construction of a percutaneous gastrostomy or jejunostomy, or through initiating parenteral nutrition.
6. Time to reintervention for persistence or recurrence of symptoms is defined as the time in days between EUS-GE/SGJ and reintervention for persistence or recurrence of symptoms of GOO (nausea, vomiting, inability to tolerate oral intake).
7. Adverse events (AEs) are specified according to the ASGE lexicon for endoscopic adverse events [2]. An adverse event is defined as "an event that prevents completion of the planned procedure and/or results in admission to hospital, prolongation of existing hospital stay, another procedure (needing sedation/anesthesia), or subsequent medical consultation." Severity of AEs is graded according to the Clavien-Dindo Complication Score (severe is defined as ≥ 3B) [3, 4].
8. Quality of life will be measured by two cancer specific questionnaires (core-questionnaire EORTC QLQ-C30 supplemented with a disease-specific module EORTC QLQ-STO22 focusing on gastric complaints) to measure health related quality of life of cancer patients [5, 6]. In addition, the EQ-5D-3L questionnaire is used in cost-effectiveness analysis [7].

9. Time to start chemotherapy is defined as the number of days after EUS-GE/SGJ until chemotherapy is started (if applicable).
10. Length of hospital stay is defined as days of hospitalization between EUS-GE/SGJ and hospital discharge. If patients are transferred back to a referring hospital, the final date of discharge from their referring hospital will be registered.
11. Readmission: number and duration of hospital readmissions within 30 days after EUS-GE/SGJ.
12. Weight is defined as patients' weight in kilograms. Comparison is made between weight at baseline and weight one month after EUS-GE/SGJ.
13. Survival is defined by the number of days after EUS-GE/SGJ until death. The cause of death will be registered.
14. Costs are defined as the intramural costs that were involved with EUS-GE/SGJ, collected from the electronic hospital records and linked to the Dutch unit costs. Primary outcome measures from our economic evaluation are Quality Adjusted Life Years (QALY) and Incremental Cost Effectiveness Ratios (ICERs).

References

1. Adler DG, Baron TH. Endoscopic palliation of malignant gastric outlet obstruction using self-expanding metal stents: experience in 36 patients. Am J Gastroenterol. 2002;97:72–8.

2. Cotton PB, Eisen GM, Aabakken L, Baron TH, Hutter MM, Jacobson BC, et al. A lexicon for endoscopic adverse events: report of an ASGE workshop. Gastrointest Endosc. 2010;71:446–54.

3. Clavien PA, Sanabria JR, Strasberg SM. Proposed classification of complications of surgery with examples of utility in cholecystectomy. Surgery. 1992;111:518–26.

4. Dindo D, Demartines N, Clavien P-A. Classification of Surgical Complications. Ann Surg. 2004;240:205–13.

5. Aaronson NK, Ahmedzai S, Bergman B, Bullinger M, Cull A, Duez NJ, et al. The European Organization for Research and Treatment of Cancer QLQ-C30: A Quality-of-Life Instrument for Use in International Clinical Trials in Oncology. JNCI Journal of the National Cancer Institute. 1993;85:365–76.

6. Vickery CW, Blazeby JM, Conroy T, Arraras J, Sezer O, Koller M, et al. Development of an EORTC disease-specific quality of life module for use in patients with gastric cancer. Eur J Cancer. 2001;37:966–71.

7. Rabin R, Charro F de. EQ-5D: a measure of health status from the EuroQol Group. Ann Med. 2001;33:337–43.
